# Hepaticocholecystic duct: A pitfall of cholecystectomy

**DOI:** 10.1002/ccr3.1875

**Published:** 2018-11-14

**Authors:** Andrea Laurenzi, Marc‐Antoine Allard, Eric Vibert

**Affiliations:** ^1^ Centre Hépato‐Biliaire Hôpital Paul Brousse Villejuif France

**Keywords:** accessory bile duct, anatomical variation, bile duct injury, biliary anatomy, cholecystectomy, Luschka duct

## Abstract

The anatomical variations of accessory biliary ducts account for up to 2% of the population. The two types of ducts are the: subvescical and hepaticocholecystic. The knowledge of such variations is extremely important during cholecystectomy in order to avoid possible postoperative complications such as biliary injury or choleperitoneum.

The image shows a liver graft (Figure 1A) on which we discovered an hepaticocholecystic duct during cholecystectomy. The duct has been tied and graft implanted without technical variations. The magnetic resonance cholangiopancreatography (MRCP) (Figure 1B) realized postoperatively, showed the ligated duct which is in communication with the biliary tree. No clinical consequences have been observed.

**Figure 1:**
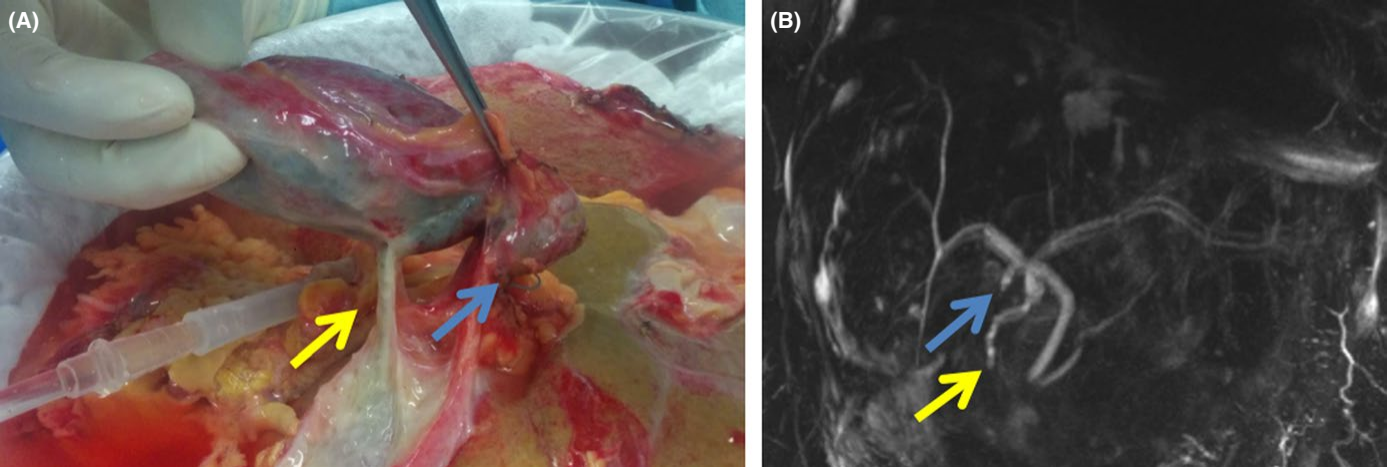
A, Cholecystectomy on liver graft discovering the hepaticocholecystic duct (yellow arrow) and cystic duct (blue arrow). B, MRCP showing the hepaticocholecystic duct tied (yellow arrow) and the cystic duct (blue arrow)

The term duct of Luschka is used to describe variations to biliary anatomy concerning the gallbladder or its bed found during cholecystectomy. The eponym Luschka refers, in a wrong way, to Hubert von Luschka that described glands on the biliary wall, never mentioning accessory biliary ducts.^1^ A recent review of the literature has reclassified the accessory biliary ducts in two subgroups: the subvescical and the hepaticocholecystic ducts.^2^

The accessory biliary ducts “subvescical” (segmental or accessory) are located under the gallbladder bed and they are not in communication with the gallbladder. On the contrary, the ducts known as “hepaticocholecystic” (proper or aberrant) drain a part of the liver directly into the gallbladder. This explains why if the duct is not individualized during cholecystectomy a lesion of an hepaticocholecystic duct can lead to a choleperitoneum in the postoperative period. The ligation of the duct leads to an atrophy of the drained area, which usually remains asymptomatic.

## CONFLICT OF INTEREST

None declared.

## AUTHOR CONTRIBUTIONS

Andrea Laurenzi: conceived the work and drafted the manuscript. Marc Antoine Allard: contributed to critical revision and final approval. Eric Vibert: conceived the work and contributed to final approval.
